# Quality of anticoagulation and outcomes after mechanical aortic valve replacement in patients with atrial fibrillation: a nationwide cohort study

**DOI:** 10.1093/ehjqcco/qcaf028

**Published:** 2025-05-14

**Authors:** Joonas Lehto, Rikhard Björn, Olli Halminen, Miika Linna, Jari Haukka, Jukka Putaala, Pirjo Mustonen, Janne Kinnunen, Juha Hartikainen, Juhani K E Airaksinen, Tuomas O Kiviniemi, Mika Lehto

**Affiliations:** Heart Center, Turku University Hospital and University of Turku, POB 52, Turku FI-20521, Finland; Heart Center, Turku University Hospital and University of Turku, POB 52, Turku FI-20521, Finland; Department of Industrial Engineering and Management, Aalto University, Espoo, Finland; Department of Industrial Engineering and Management, Aalto University, Espoo, Finland; Heart Center, Kuopio University Hospital and University of Eastern Finland, Kuopio, Finland; Faculty of Medicine, University of Helsinki, Helsinki, Finland; Department of Neurology, Helsinki University Hospital, University of Helsinki, Helsinki, Finland; Heart Center, Turku University Hospital and University of Turku, POB 52, Turku FI-20521, Finland; Department of Neurology, Helsinki University Hospital, University of Helsinki, Helsinki, Finland; Heart Center, Kuopio University Hospital and University of Eastern Finland, Kuopio, Finland; Heart Center, Turku University Hospital and University of Turku, POB 52, Turku FI-20521, Finland; Heart Center, Turku University Hospital and University of Turku, POB 52, Turku FI-20521, Finland; Faculty of Medicine, University of Helsinki, Helsinki, Finland; Heart and Lung Center, Helsinki University Hospital and University of Helsinki, Helsinki, Finland

**Keywords:** Aortic valve replacement, Atrial fibrillation, Bleeding, Stroke, Acute coronary syndrome

## Abstract

**Aims:**

Mechanical aortic valve replacement (AVR) remains the primary treatment for younger patients with severe aortic valve disease. However, limited information is available regarding the quality of the required lifelong vitamin K antagonist (VKA) therapy, atrial fibrillation (AF), and their relationship with adverse events after AVR. This study assessed the quality of VKA therapy prior to bleeding and ischaemic events following mechanical AVR in patients with AF.

**Methods and results:**

The registry-based Finnish AntiCoagulation in Atrial Fibrillation study combining data from several Finnish healthcare registers covers all patients diagnosed with AF during 2007–18 in Finland. This analysis included patients undergoing mechanical AVR before or after the AF diagnosis. A total of 1086 patients with mechanical AVR and AF either before (41.2%) or after (58.8%) the operation were identified. Cumulative incidence estimates at 10 years after AVR were 27.9% for significant bleeding, 5.8% for intracranial haemorrhage, 12.8% for ischaemic stroke, and 7.2% for myocardial infarction. Time in therapeutic range (TTR) < 80% with international normalized ratio (INR) target 2.0–3.5 was associated with higher bleeding occurrence [adjusted hazard ratio (aHR) 1.97, 1.39–2.79, *P* < 0.001]. Time in therapeutic range with INR target ≥2.0 was associated with higher stroke occurrence (aHR/standard deviation 1.22, 1.01–1.46, *P* = 0.035). Mortality was high (28.9%/10 years), and TTR <80% was associated with higher mortality (aHR 2.74, 2.00–3.76, *P* < 0.001).

**Conclusion:**

Adverse events, particularly major bleeding, are common in patients with AF following mechanical AVR, and mortality is high. Suboptimal TTR appears to predict bleeding episodes, ischaemic stroke, and death, and it could be useful in high-risk patient identification and targeting of preventive strategies.

**Trial registration:**

Finnish AntiCoagulation in Atrial Fibrillation study, ClinicalTrials Identifier: NCT04645537, https://clinicaltrials.gov/ct2/show/NCT04645537

## Graphical Abstract

**Graphical Abstract:**
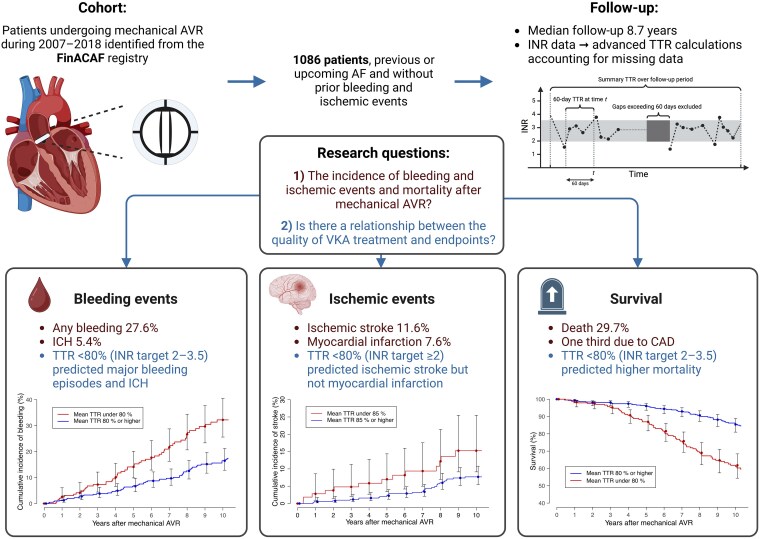

Key Learning Points What is already known:

- The reported incidences of ischaemic and bleeding events following mechanical aortic valve replacement (AVR) have remained high, even though most patients selected for the procedure are deemed to have a low risk of thromboembolic and bleeding complications.
- Existing data on anticoagulation quality during complications are limited and often troubled by inadequate consideration of missing international normalized ratio data or otherwise oversimplified time in therapeutic range (TTR) calculations.
- There is a lack of large-scale epidemiological studies addressing the combined thromboembolic risk of atrial fibrillation (AF) and mechanical aortic valve prosthesis.

What this study adds:

- In the Finnish AntiCoagulation in Atrial Fibrillation registry, which included 1086 patients with mechanical AVR and either prior or impending AF, but no history of bleeding or ischaemic events, suboptimal TTR appeared to predict bleeding episodes, ischaemic stroke, and death.
- Time in therapeutic range calculations could serve as a valuable tool for risk prediction after mechanical AVR, enabling more effective targeting of preventive measures.
- Atrial fibrillation substantially increases thromboembolic risk in patient with mechanical AVR, underscoring the importance of optimized vitamin K antagonist therapy in this high-risk population.

## Introduction

Aortic valve stenosis and regurgitation account for the majority of valvular diseases requiring intervention. The advent of transcatheter aortic valve replacement has expanded the use of biological valve replacements to younger patients. However, mechanical aortic valve replacement (AVR) remains the preferred option for patients under 50 years of age and selected patients aged 50–65 years.^1,2^ While direct oral anticoagulants have largely replaced vitamin K antagonist (VKA) therapy as the standard anticoagulation for patients with non-valvular atrial fibrillation (AF), individuals with mechanical valves continue to require lifelong VKA therapy, managed through international normalized ratio (INR) monitoring, due to the thrombogenic nature of the valve prosthesis. Despite careful INR-guided VKA therapy, deviations from the therapeutic range are common due to various reasons, such as poor adherence and physiological changes due to diet, concurrent medication, comorbidities, and ageing. The reported incidences of ischaemic and bleeding events following mechanical AVR remain high,^3–6^ even though most patients selected for mechanical valves are deemed to have a low risk of thromboembolic and bleeding complications. Although these complications are often attributed to suboptimal VKA therapy, data on the quality of anticoagulation during such events are limited. Furthermore, while the majority of patients with mechanical AVR have either pre-existing AF or develop AF postoperatively,^7^ large-scale epidemiological studies on their combined thromboembolic risk are lacking, and even the benefit of an elevated INR goal in these patients remains uncertain.^8^

Our study aimed to provide a comprehensive analysis of the quality of VKA therapy in patients with AF after mechanical AVR and its association with ischaemic and bleeding events during long-term follow-up. To achieve this aim, we utilized nationwide clinical and laboratory data and employed more advanced time in therapeutic range (TTR) calculations accounting for missing data. In addition, we sought to evaluate the occurrence of ischaemic events, bleeding episodes, and mortality in this patient population, as well as the effect of AF on thromboembolic risk after mechanical AVR.

## Methods

### Study population

The Finnish AntiCoagulation in Atrial Fibrillation study (ClinicalTrials Identifier: NCT04645537; ENCePP Identifier: EUPAS29845) is a retrospective, nationwide, registry-based cohort study encompassing all patients diagnosed with AF in Finland between 2004 and 2008.^9^ The inclusion criterion for the original cohort was an International Classification of Diseases, Tenth Revision (ICD-10), diagnosis code I48, representing AF or flutter (collectively referred to as AF). Cohort entry was defined as the date of the first recorded AF diagnosis. Patients with AF were identified using data from the following national healthcare registers: hospitalizations and outpatient specialist visits (HILMO) registry, the primary health care (AvoHILMO) registry, and the National Reimbursement Register upheld by the Social Insurance Institute (KELA) registry. General exclusion criteria included age below 20 years at the time of AF diagnosis and permanent migration abroad before 31 December 2018. This substudy was conducted using a cohort of patients who underwent either isolated or concomitant mechanical AVR, both before or after the AF diagnosis and cohort entry. Follow-up commenced on the date of the mechanical AVR procedure (on or after 1 January 2004) and concluded on 31 December 2018. The patient selection process is outlined in the study flowchart (see Supplementary material online, *Figure S1*). To ensure the analysis focused on first-ever outcome events, patients with a history of clinically significant bleeding, ischaemic stroke, or myocardial infarction (MI) before the index operation were excluded. The definitions of the comorbidities are detailed in Supplementary material online, *Table S1*.

The patients’ individual highest annual taxable income (in 1000-euro accuracy) during 2004–18 was derived from the national Tax Register. The highest achieved educational level categorized according to the International Standard Classification of Education (ISCED) was obtained from Statistics Finland. Educational level was divided into three categories: Category 1: ISCED 0–2 (no registered education, preprimary, primary, or lower secondary educations); Category 2: ISCED 3–4 (upper secondary, vocational, or post-secondary non-tertiary education); and Category 3: ISCED 5–8 (tertiary, bachelor’s level, master’s level, or doctoral level education).

### Time in therapeutic range analyses

Time in therapeutic range was continuously calculated using the Rosendaal method^10^ based on INR values recorded over the preceding 60 days. If the interval between consecutive INR measurements exceeded 60 days, the most recent INR value was carried forward, and any periods exceeding 60 days from the previous measurement were excluded from the analysis. This methodology is described in detail in a previous study.^11^ To minimize bias from missing data, the effect of TTR was assessed in patients with average ≥6 INR measurements per follow-up year or a total of ≥20 measurements prior to the event of interest, death, or censoring, with a minimum follow-up period of 60 days. Only TTR values recorded ≥60 days after the surgery were included. An INR target of 2.0–3.5 was used in the calculations unless stated otherwise. The longitudinal quality of VKA therapy was then evaluated using the mean of the calculable 60-day TTR values throughout the follow-up period. For the analyses of ischaemic and bleeding events, only TTR values obtained prior to the event were considered.

### Study outcomes

Outcome events of interest included clinically significant bleeding, intracranial bleeding, gastrointestinal (GI) bleeding, MI, and ischaemic stroke. The diagnoses are outlined in Supplementary material online, *Table S1*. For composite endpoint analyses, clinically significant bleeding and ischaemic stroke were combined. The outcome was considered to occur on the date of the first recorded event of interest following the index surgery. Only diagnoses from the hospital register were included to ensure that the event was both major and clinically relevant. In the TTR and INR analyses, patients who experienced the endpoint in question within a 30-day postoperative blanking period were excluded.

### Statistical analysis

CHA_2_DS_2_-VA score was calculated similar to CHA_2_DS_2_-VASc score^12^ but with the omission of female sex.^13^ Univariable competing risk analysis was performed using the Fine–Gray subdistribution hazard model to estimate the association between baseline characteristics and outcomes, excluding mortality. Baseline differences between patients with TTR <80 and ≥80% were assessed using the Mann–Whitney test and χ^2^ test. In the multivariable Fine–Gray subdistribution hazard model analysing the association between TTR and outcomes (other than all-cause mortality), adjustments were made for established risk factor for ischaemic and bleeding events, including age, sex, preoperative heart failure, hypertension, diabetes, vascular disease, alcohol abuse, renal failure, and liver cirrhosis or failure. Mortality analyses were performed using Cox proportional hazards regression univariable model and multivariable model with adjustments similar to those in the Fine–Gray model. The proportional hazards assumption was assessed using Schoenfeld residuals. Predictive validity was assessed using the time-dependent area under the receiver operating characteristics (ROC) curve. Changes over time and across groups were tested using univariable unstructured linear mixed model (LMM) for repeated measures. Statistical analyses were performed using R software version 4.1.1 (R Foundation for Statistical Computing, Vienna, Australia). Graphs were drawn using R and BioRender (BioRender.com).

### Study ethics

The study protocol was approved by the Ethics Committee of the Medical Faculty of Helsinki University, Helsinki, Finland (reference number 15/2017), and granted research permission from the Helsinki University Hospital (HUS/46/2018). Respective permissions were obtained from the Finnish register holders (KELA 138/522/2018; THL 2101/5.05.00/2018; Population Register Centre VRK/1291/2019-3, and Tax Register VH/874/07.01.03/2019). Patient identification numbers were pseudonymized, and the research team received individualized, but de-identified data. Informed consent was waived due to the retrospective registry-based nature of the study. The study conforms to the Declaration of Helsinki, as revised in 2002.

## Results

Out of 410 102 patients with AF, we identified 1086 patients {27.1% female, median age 62.8 [inter-quartile range (IQR) 56.2–68.3] years} who underwent mechanical AVR during the study period. Most of the AVR procedures occurred prior to the AF diagnosis (58.8%). Indication for the operation was isolated aortic valve stenosis in 406 (37.4%) patients, isolated aortic valve insufficiency in 215 (19.8%) patients, and mixed aortic valve disease in 438 (40.3%) patients. In addition, active or previous endocarditis was identified in 59 (5.4%) patients. The median follow-up time was 8.7 (IQR 4.8–12.0) years. During the follow-up, a median of 54 (IQR 4–149) INR measurements per patient were available. Comprehensive INR data (average ≥6 INR measurements per follow-up year or ≥20 measurements overall) were available for 675 (64.5%) patients. In these patients, the mean TTR (INR target 2.0–3.5) during follow-up was 80.8 ± 15.5%. International normalized ratio and TTR values are illustrated in the Supplementary material (Supplementary material online, *Figures S2* and *S3*).

### Composite endpoint receiver operating characteristics analysis

During the follow-up period, the composite endpoint of clinically significant bleeding or ischaemic stroke occurred in 374 patients (34.4%). Median time from AVR to endpoint was 4.2 years (IQR 1.7–7.8 years). The patients with composite endpoint were older and had more often vascular disease and diabetes (see Supplementary material online, *Table S2*). No major differences were found in the number of patients with comprehensive INR data available when stratified by endpoints (see Supplementary material online, *Table S3*). At 10-year follow-up, the ROC analysis yielded area under curve of 61.5% [95% confidence interval (CI) 54.8–68.1%] with Youden’s index optimum TTR cut-off point of 80.5% resulting in positive predictive value of 50.1% and negative predictive value of 77.5%. Multivariable competing risk analysis identified TTR <80% to be associated with higher composite endpoint occurrence (aHR 1.83, 95% CI 1.32–2.53, *P* < 0.001, Supplementary material online, *Figure S4*). Patients with TTR <80% represented less aortic valve insufficiency, more previous endocarditis, and marginal differences in CHA_2_DS_2_-VA score (see Supplementary material online, *Table S4*).

### Bleeding events

**Figure 1:**
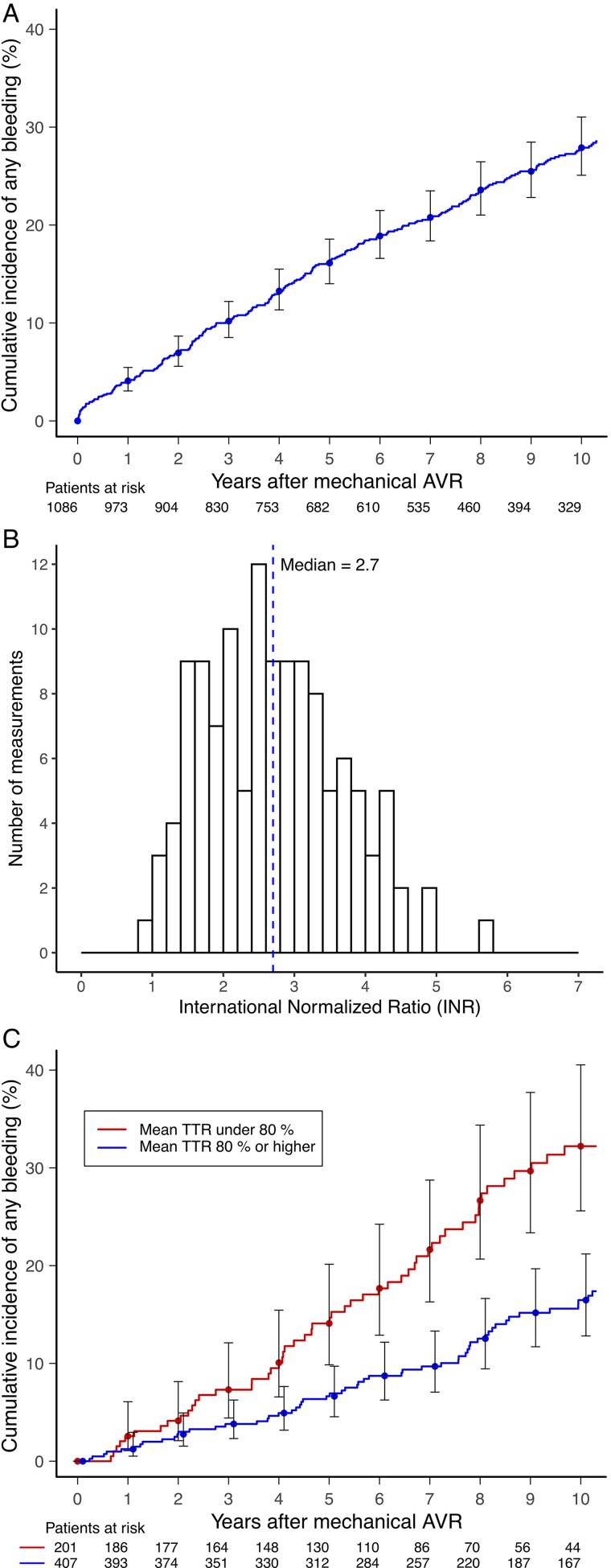
Cumulative incidence of any significant bleeding after mechanical aortic valve replacement (*A*), international normalized ratio at the time of the event (*B*), and the unadjusted cumulative incidence stratified by time in therapeutic range (international normalized ratio target 2.0–3.5) < 80 and ≥80% (*C*).

During the follow-up, 300 (27.6%) patients experienced a significant bleeding event, of whom 59 had intracranial haemorrhages (ICHs) and 128 had GI bleeding episodes. The median time between AVR and the first bleeding event was 4.5 (IQR 2.0–8.0) years (*Figure 1A*). Patients with bleedings were older and had a higher prevalence of diabetes compared with their peers without bleedings (*Table 1*). The cumulative incidence of clinically significant bleeding was 3.8 per 100 patient-years (*Table 2*) and highest in patients with CHA_2_DS_2_-VA score ≥5 (*Table 3*). The INR value at the time of bleeding event was available for 128 (44.6%) patients. The median INR was 2.7 (IQR 2.0–3.5), and a supratherapeutic value of >3.5 was found in 30 (23.4%) patients (*Figure 1B*).

**Table 1:** Baseline characteristics of patients with and without significant bleeding event after mechanical aortic valve replacement and univariable Fine–Gray subdistribution hazard ratios

	No bleeding *n* = 786	Bleeding event *n* = 300	Hazard ratio (95% CI)	*P*-value
Age, years	61.8 [55.2–67.6]	65.0 [58.9–69.8]	1.18 (1.05–1.33)	0.006
Year of index surgery	2008 [2005–12]	2007 [2005–9]	0.93 (0.81–1.07)	0.320
Female sex	219 (27.9)	75 (25.0)	0.81 (0.63–1.05)	0.110
Aortic valve stenosis	620 (78.9)	224 (74.7)	0.83 (0.65–1.08)	0.160
Aortic valve insufficiency	474 (60.3)	179 (59.7)	1.03 (0.81–1.29)	0.830
Previous endocarditis	47 (6.0)	12 (4.0)	0.81 (0.45–1.47)	0.490
Any vascular disease	201 (25.6)	94 (31.3)	1.24 (0.97–1.58)	0.089
Coronary artery disease	193 (24.6)	88 (29.3)	1.2 (0.94–1.53)	0.150
Diabetes	188 (23.9)	86 (28.7)	1.49 (1.16–1.92)	0.002
Dyslipidaemia	391 (49.7)	156 (52.0)	1.08 (0.86–1.36)	0.490
Heart failure	176 (22.4)	75 (25.0)	1.14 (0.88–1.48)	0.320
Hypertension	645 (82.1)	246 (82.0)	1.12 (0.84– 1.5)	0.440
Prior TIA	25 (3.2)	11 (3.7)	1.56 (0.83–2.95)	0.170
Abnormal liver function	4 (0.5)	1 (0.3)	1.66 (0.22–12.3)	0.620
Abnormal renal function	4 (0.5)	1 (0.3)	0.62 (0.085–4.59)	0.640
Alcohol use disorder	16 (2.0)	6 (2.0)	1.29 (0.59–2.78)	0.520
Psychiatric disorder	50 (6.4)	12 (4.0)	0.89 (0.51–1.55)	0.680
Modified HAS-BLED score	1.0 [1.0–2.0]	1.0 [1.0–2.0]	1.12 (1–1.25)	0.051
CHA_2_DS_2_-VA score	2.0 [1.0–3.0]	2.0 [1.0–3.0]	1.21 (1.08–1.35)	<0.001

Values denote *n* (%) or median [25th—75th percentile]. Standardized hazard ratio for continuous variables.

CHA_2_DS_2_-VA, congestive heart failure, hypertension, age ≥75 years, diabetes, history of stroke, or TIA, vascular disease, age 65–74 years; CI, confidence interval; modified HAS-BLED score, hypertension, abnormal renal or liver function, prior stroke, bleeding history, age >65 years, alcohol abuse, concomitant antiplatelet/NSAIDs (no labile INR, maximum score 8); TIA, transient ischaemic attack.

**Table 2:** Cumulative incidence estimates and incidence rates of clinically significant bleeding, intracranial haemorrhage, ischaemic stroke, myocardial infarction, and death after mechanical aortic valve replacement

	30 days	1 year	5 years	10 years	Incidence rate (per 100 patient-years)^a^
Clinically significant bleeding	1.2% (0.7–2.0%)	4.1% (3.0–5.4%)	16.1% (13.9–18.5%)	27.9% (25.0–30.9%)	3.8 (3.3–4.2)
ICH	0.1% (0.0–0.5%)	0.6% (0.2–1.2%)	2.7% (1.8–3.8%)	5.8% (4.4–7.5%)	0.7 (0.5–0.8)
Ischaemic stroke	1.1% (0.6–1.9%)	2.6% (1.8–3.7%)	6.4% (5.0–8.1%)	12.8% (10.7–8.1%)	1.4 (1.1–1.6)
MI	0.6% (0.3–1.3%)	1.7% (1.0–2.6%)	3.2% (2.2–4.4%)	7.2% (5.6–9.1%)	0.9 (0.7–1.1)
Death	2.6% (1.6–3.5%)	4.8% (3.5–6.1%)	13.5% (11.4–15.6%)	28.9% (25.9–31.9%)	3.3 (2.9–3.7)

Values denote cumulative incidence estimates with death as a competing event in major bleeding, ICH, ischaemic stroke, and MI events. Values in parentheses are 95% confidence intervals. Mortality estimated with Kaplan–Meier method.

ICH, intracranial haemorrhage; MI, myocardial infarction.

^a^After 30-day postoperative period.

**Table 3:** The incidence rates (per 100 patient-years) of clinically significant bleeding episodes, intracranial haemorrhages, and ischaemic strokes after mechanical aortic valve replacement stratified by CHA_2_DS_2_-VA score

CHA_2_DS_2_-VA	0 *n* = 99	1 *n* = 275	2 *n* = 328	3 *n* = 214	4 *n* = 119	≥5 *n* = 51
Clinically significant bleeding	3.1 (2.0–4.4)	2.3 (1.7–2.9)	3.5 (2.8–4.3)	5.6 (4.4–6.8)	6.1 (4.2–8.1)	6.3 (3.3–9.6)
ICH	0.7 (0.2–1.2)	0.4 (0.2–0.7)	0.6 (0.2–1.0)	1.1 (0.6–1.7)	0.6 (0.1–1.3)	0.7 (0.0–1.7)
Ischaemic stroke	1.0 (0.5–1.7)	0.7 (0.4–1.1)	1.2 (0.8–1.6)	2.2 (1.4–3.0)	2.5 (1.4–3.8)	2.6 (0.7–4.8)

Events during 30-day postoperative period excluded.

Values denote incidence rates (per 100 patient-years). Values in parentheses are 95% confidence intervals.

CHA_2_DS_2_-VA, congestive heart failure, hypertension, age ≥75 years, diabetes, history of stroke or TIA, vascular disease, age 65–74 years; ICH, intracranial haemorrhage.

Time in therapeutic range for the 60 days preceding the first bleeding event could be calculated for 114/287 (39.7%) events. The median TTR was 79.0% (IQR 59.8–96.7%). No significant linear correlation was observed between time and INR values during the 60 days preceding the bleeding event (fixed-effects estimate +0.124/60 days, *P* = 0.464, Supplementary material online, *Figure S5B*). Multivariable competing risk analysis identified TTR <80% to be associated with increased bleeding risk (aHR 1.97, 95% CI 1.39–2.79, *P* < 0.001) (*Figure 1C*). The long-term TTR in patients with a bleeding episode was 78.8 ± 16.7% compared with 82.5 ± 14.4% in those without.

### Intracranial bleeding

No clinically meaningful differences were found between patients with and without ICH (see Supplementary material online, *Table S5*). International normalized ratio at the time of ICH event was available for 37 (63.8%) patients. The median INR was 2.9 (IQR 1.8–3.5), and a supratherapeutic value of >3.5 was found in 9 (24.3%) patients. The 60-day TTR before ICH could be calculated in 26/58 (44.8%) patients. Although no significant correlation between time and INR values before ICH was found in the LMM analysis (fixed-effects estimate −0.425/60 days, *P* = 0.137), a substantial amount of the preceding INR values before ICH were at supratherapeutic level (see Supplementary material online, *Figure S5C*), and the median 60-day TTR before ICH was 67.2% (IQR 41.1–81.5%). The multivariable competing risk analysis identified TTR <80% to be associated with increased ICH risk (aHR 2.41, 95% CI 1.18–4.93, *P* = 0.016) (see Supplementary material online, *Figure S6*).

### Ischaemic stroke

**Figure 2:**
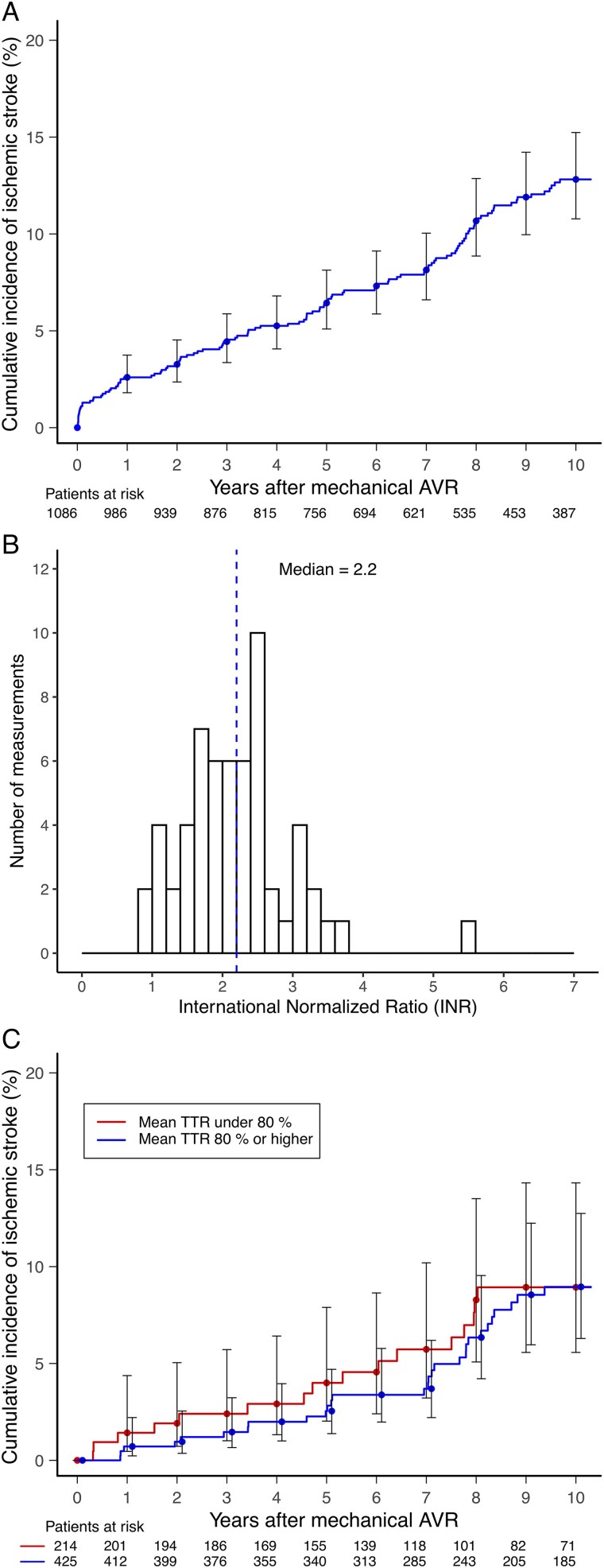
Cumulative incidence of ischaemic stroke after mechanical aortic valve replacement (*A*), international normalized ratio at the time of the event (*B*), and the unadjusted cumulative incidence stratified by mean time in therapeutic range (international normalized ratio target 2.0–3.5) < 80 and ≥80% (*C*).

During the follow-up period, 126 (11.6%) ischaemic stroke episodes were detected. The median time between AVR and stroke was 4.9 (IQR 1.7–7.8) years (*Figure 2A*). Baseline characteristics of patients with and without stroke are detailed in *Table 4*. The INR value at the time of ischaemic stroke was available for 59 (46.8%) patients. The median INR was 2.2 (IQR 1.7–2.6), and a subtherapeutic value of <2.0 was found in 20 (33.9%) patients (*Figure 2B*).

**Table 4:** Baseline characteristics of patients with and without ischaemic stroke after mechanical aortic valve replacement and univariable Fine–Gray subdistribution hazard ratios

	No ischaemic stroke *n* = 960	Ischaemic stroke *n* = 126	Hazard ratio (95% CI)	*P*-value
Age, years	62.3 [55.6–67.8]	67.1 [59.9–73.8]	1.4 (1.11–1.78)	0.005
Year of index surgery	2008 [2005–12]	2006 [2005–9]	0.92 (0.73–1.15)	0.440
Female sex	253 (26.4)	41 (32.5)	1.26 (0.87–1.83)	0.220
Aortic valve stenosis	740 (77.1)	104 (82.5)	1.4 (0.89–2.21)	0.150
Aortic valve insufficiency	584 (60.8)	69 (54.8)	0.82 (0.58–1.16)	0.260
Previous endocarditis	50 (5.2)	9 (7.1)	1.55 (0.80–3.01)	0.200
Any vascular disease	254 (26.5)	41 (32.5)	1.26 (0.87–1.82)	0.230
Coronary artery disease	244 (25.4)	37 (29.4)	1.16 (0.79–1.71)	0.440
Diabetes	237 (24.7)	37 (29.4)	1.41 (0.96–2.07)	0.076
Dyslipidaemia	477 (49.7)	70 (55.6)	1.24 (0.88–1.77)	0.220
Heart failure	220 (22.9)	31 (24.6)	1.1 (0.74–1.65)	0.640
Hypertension	790 (82.3)	101 (80.2)	0.96 (0.63–1.48)	0.860
Prior TIA	32 (3.3)	4 (3.2)	1.22 (0.45–3.3)	0.700
Abnormal liver function	5 (0.5)	0	0.00012 (0.000043–0.00035)	<0.001
Abnormal renal function	4 (0.4)	1 (0.8)	1.65 (0.23–11.9)	0.620
Alcohol use disorder	19 (2.0)	3 (2.4)	1.41 (0.48–4.13)	0.530
Psychiatric disorder	52 (5.4)	10 (7.9)	1.95 (1.03–3.69)	0.041
Modified HAS-BLED score	1.0 [1.0–2.0]	2.0 [1.0–2.0]	1.23 (1.03–1.46)	0.022
CHA_2_DS_2_-VA score	2.0 [1.0–3.0]	3.0 [2.0–3.0]	1.35 (1.14–1.58)	<0.001

Values denote *n* (%) or median [25th—75th percentile]. Standardized hazard ratio for continuous variables.

CHA_2_DS_2_-VA, congestive heart failure, hypertension, age ≥75 years, diabetes, history of stroke or TIA, vascular disease, age 65–74 years; CI, confidence interval; modified HAS-BLED score, hypertension, abnormal renal or liver function, prior stroke, bleeding history, age >65 years, alcohol abuse, concomitant antiplatelet/NSAIDs (no labile INR, maximum score 8); TIA, transient ischaemic attack.

Time in therapeutic range during the 60 days preceding ischaemic stroke could be calculated in 45/114 (39.5%) patients. The median 60-day TTR with an INR target of ≥2.0 without an upper limit was 97.3% (IQR 84.2–100.0%). Linear mixed model analysis identified significant linear correlation between time and INR values in the 60-day period before ischaemic stroke (fixed effect estimate −0.572/60 days, *P* < 0.001) (see Supplementary material online, *Figure S5D*). No significant association between long-term TTR <80% and stroke occurrence was found with an INR target of 2.0–3.5 (aHR 1.06, 95% CI 0.60–1.86, *P* = 0.850) (*Figure 2C*). Instead, an association was found with an INR target of ≥2.0 (aHR/SD 1.22 for lower TTR, 95% CI 1.01–1.46, *P* = 0.035). The TTRs (INR target ≥2.0) in patients with and without stroke were 85.7 ± 21.9 and 90.9 ± 13.9%, respectively.

A vast majority (86.5%) of the strokes occurred after the initial AF diagnosis (see Supplementary material online, *Figure S7*). The incidence rate of stroke was 1.4 per 100 patient-years in AF and 0.9 per 100 patient-years without AF. Moreover, in 10 (7.9%) patients, the first ischaemic stroke and AF were diagnosed within 30 days of each other. Among the 45 patients with INR data available immediately before the stroke, six patients underwent a relevant invasive procedure possibly interfering the VKA therapy shortly before the stroke. The LMM analysis result remained similar after the exclusion of these patients (fixed-effects estimate −0.575/60 days, *P* < 0.001). When including all stroke patients, altogether 17/126 (13.5%) patients underwent a relevant invasive procedure within 60 days before the stroke, most of which were cardiac procedures (70.6%).

### Myocardial infarction

Overall, 82 (7.6%) MI episodes were detected during follow-up. Patients with MI were more likely to have previous vascular disease and dyslipidaemia (see Supplementary material online, *Table S6*). International normalized ratio at the time of MI was available for 33 (44.0%) patients. The median INR was 2.6 (IQR 1.8–3.0), and a subtherapeutic value of <2.0 was found in 9 (27.3%) patients. The 60-day TTR before MI could be calculated in 26 (34.7%) patients. Although no significant linear correlation between time and INR values before MI was found (fixed-effects estimate −0.443/60 days, *P* = 0.170), a notable fluctuation was detected (see Supplementary material online, *Figure S5E*), and the median 60-day TTR before MI was 72.0% (IQR 41.3–87.8%). In the multivariable competing risk analysis, no significant correlation between long-term TTR with an INR target of 2.0–3.5 (aHR 1.22, 95% CI 0.58–2.56, *P* = 0.590) or ≥2.0 (aHR/SD 1.22 for lower TTR, 95% CI 0.87–1.71, *P* = 0.260) was found (see Supplementary material online, *Figure S8*).

### Mortality

**Figure 3:**
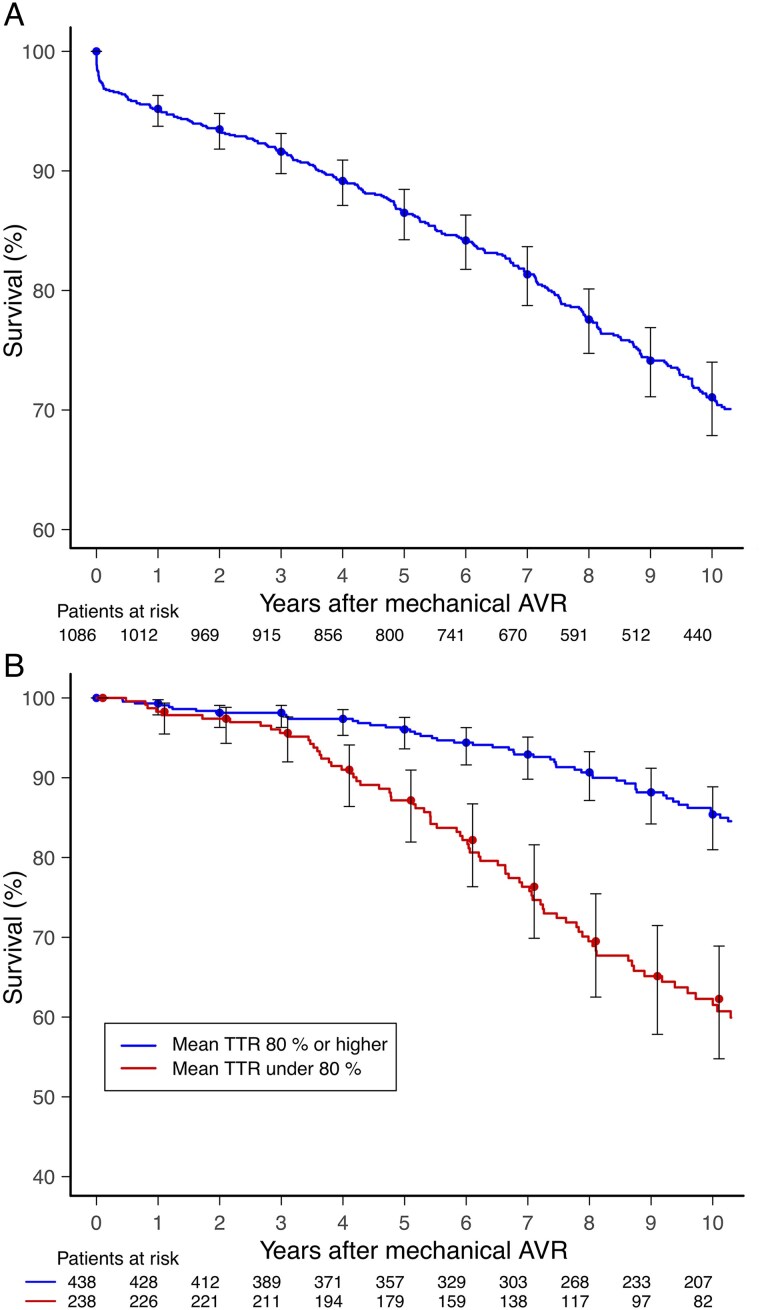
Survival after mechanical aortic valve replacement (*A*) and unadjusted survival stratified by time in therapeutic range (international normalized ratio target 2.0–3.5) < 80 and ≥80% (*B*).

During the follow-up period, 322 (29.7%) patients died. The predominant underlying diagnosis of death was coronary artery disease (CAD) (see Supplementary material online, *Table S7*). Patients who died during the follow-up were older, more frequently female, and more likely to have aortic valve stenosis, vascular disease, diabetes, dyslipidaemia, prior heart failure, hypertension, and psychiatric disorders and less likely to have aortic valve insufficiency (*Table 5*). The multivariable Cox regression analysis identified TTR <80% to be associated with higher mortality (aHR 2.74, 95% CI 2.00–3.76, *P* < 0.001) (*Figure 3*). The TTRs in patients deceased or surviving the study period were 73.7 ± 18.3 and 82.9 ± 13.9%, respectively.

**Table 5:** Baseline characteristics of patients alive and deceased at the end of follow-up after mechanical aortic valve replacement and univariable Cox regression hazard ratios

	Alive *n* = 764	Deceased *n* = 322	Hazard ratio (95% CI)	*P*-value
Age, years	60.8 [54.3–66.4]	67.7 [62.0–73.6]	2.04 (1.78–2.33)	<0.001
Year of index surgery	2009 [2006–12]	2006 [2005–8]	0.95 (0.8–1.12)	0.520
Female sex	187 (24.5)	107 (33.2)	1.3 (1.03–1.64)	0.026
Aortic valve stenosis	581 (76.0)	263 (81.7)	1.43 (1.08–1.9)	0.013
Aortic valve insufficiency	479 (62.7)	174 (54.0)	0.79 (0.63–0.98)	0.032
Previous endocarditis	41 (5.4)	18 (5.6)	1.36 (0.84–2.18)	0.209
Any vascular disease	171 (22.4)	124 (38.5)	1.91 (1.53–2.39)	<0.001
Coronary artery disease	164 (21.5)	117 (36.3)	1.87 (1.49–2.34)	<0.001
Diabetes	179 (23.4)	95 (29.5)	1.7 (1.34–2.17)	<0.001
Dyslipidaemia	372 (48.7)	175 (54.3)	1.27 (1.02–1.58)	0.036
Heart failure	153 (20.0)	98 (30.4)	1.73 (1.36–2.2)	<0.001
Hypertension	610 (79.8)	281 (87.3)	1.95 (1.41–2.71)	<0.001
Prior TIA	30 (3.9)	6 (1.9)	0.75 (0.33–1.67)	0.477
Abnormal liver function	4 (0.5)	1 (0.3)	1.6 (0.22–11.44)	0.638
Abnormal renal function	2 (0.3)	3 (0.9)	2.76 (0.89–8.61)	0.080
Alcohol use disorder	17 (2.2)	5 (1.6)	1.09 (0.45–2.63)	0.854
Psychiatric disorder	43 (5.6)	19 (5.9)	1.76 (1.1–2.8)	0.018
Modified HAS-BLED score	1.0 [1.0–2.0]	2.0 [1.0–2.0]	1.52 (1.36–1.7)	<0.001
CHA_2_DS_2_-VA score	2.0 [1.0–3.0]	3.0 [2.0–4.0]	1.73 (1.57–1.91)	<0.001

Values denote *n* (%) or median [25th—75th percentile]. Standardized hazard ratio for continuous variables.

CHA_2_DS_2_-VA, congestive heart failure, hypertension, age ≥75 years, diabetes, history of stroke or TIA, vascular disease, age 65–74 years; CI, confidence interval; modified HAS-BLED score, hypertension, abnormal renal or liver function, prior stroke, bleeding history, age >65 years, alcohol abuse, concomitant antiplatelet/NSAIDs (no labile INR, maximum score 8); TIA, transient ischaemic attack.

## Discussion

### Main findings

The main findings of this study are as follows: (i) adverse events, especially bleeding episodes, are common after mechanical AVR in patients with AF; (ii) suboptimal mean TTR appears to identify patients at high risk of both ischaemic and bleeding events; and (iii) mortality is substantial among patients with AF after mechanical AVR, with a worse prognosis in those with low TTR.

### Bleeding events

In this study, clinically significant bleeding episodes were twice as common as ischaemic strokes, with over a quarter of patients experiencing a bleeding event within 10 years after the surgery. The bleeding rates observed are nearly identical to those previously reported,^5,6^ though lower rates have also been noted,^3,4^ likely due to underdiagnosing. Consistent with the present results, earlier studies have reported higher rates of major bleeding compared with major strokes. A majority of the bleeding events were GI. Although the incidence of ICHs was roughly half that of strokes, ICHs are typically weighted more heavily than strokes in net clinical benefit analyses of oral anticoagulation. As a result, the current ratio of bleeding to ischaemic events seems imbalanced, suggesting that greater emphasis should be placed on preventing major bleeding episodes. Considering the high bleeding risk and the limited evidence to support the higher INR target in patients with AF after mechanical AVR,^8^ preferring lower INR target (median INR 2.5) could be reasonable at least in younger patients with AF without additional thromboembolic risk factors.

Low long-term TTR prior to the event, with an INR target of 2–3.5, was significantly associated with a higher occurrence of both ICHs and general bleeding episodes. This finding aligns with previous reports,^14,15^ although statistical significance was not reached in all prior studies.^16^ In addition, short-term lability of INR values seems to precede bleeding events, particularly ICHs. Identifying these high-risk patients may provide an opportunity to address the INR fluctuations, potentially reducing the risk of bleeding.

Anticoagulation therapy following mechanical valve surgery has primarily focused on the prevention of thromboembolic events. This is reflected in the fact that, aside from recommendations for initial valve prosthesis selection, operative approach, and periprocedural antithrombotic treatment, neither the European nor American guidelines address the prevention of severe bleeding episodes.^1,2^ While the current antithrombotic approach appears effective in preventing strokes when properly performed, there is a clear need for effective expedients to reduce the significant bleeding burden.

### Ischaemic events

In our study, approximately one in eight patients experienced an ischaemic stroke within 10 years after the surgery. Prior studies have reported stroke incidences of 3–4% within 5 years and 5–7% within 10 years after mechanical AVR.^3–6^ Our data revealed a notably higher incidence of stroke, likely reflecting the additional thrombotic risk associated with AF. This hypothesis is supported by the fact that the majority of strokes occurred after the diagnosis of AF, both in our study and in a previous report,^6^ with a significantly higher stroke rate during AF. When stratified by CHA_2_DS_2_-VA score (*Table 3*), the rate of ischaemic strokes was equal to or higher in the low-to-intermediate-risk patients (CHA_2_DS_2_-VA ≤1 point) compared with patients with AF without anticoagulation therapy in general,^12^ whereas high-risk patients exhibited a lower relative risk. Among the low-to-intermediate-risk patients, a substantial proportion (54.5%) had INR values below the therapeutic range at the time of stroke. In contrary, only a small fraction of low-to-intermediate-risk patients had supratherapeutic INR at the time of bleeding events (12.5% for ICH and 23.3% for any clinically significant bleeding). Overall, these findings suggest that adequate VKA therapy is effective in stroke prevention, while poor treatment adherence is associated with ischaemic strokes, particularly in low-to-moderate-risk patients. Therefore, although the incidence rate of stroke is markedly higher during AF, the focus should be on improving TTR rather than raising the INR target, at least in low-to-moderate-risk patients. Furthermore, given the elevated thromboembolic risk in patients with mechanical AVR with concomitant AF, greater emphasis should be placed on managing lifestyle and metabolic risk factors to prevent AF in this population.^17^

Low TTR correlated with a higher occurrence of stroke only when the INR target was 2 or higher. Therefore, while bleeding episodes can be predicted by identifying patients with labile INR values, these patients are not necessarily at the highest risk of stroke. Instead, patients with occasional deviations below the target range are at the highest risk of stroke, most likely due to their tendency to forget their daily VKA doses. While the association between low TTR and thromboembolic events in patients with AF is well established,^18,19^ data on patients with mechanical AVR are limited. In an Italian study, TTR was calculated for the last year of follow-up, resulting in a questionable timely relation between TTR and thromboembolic events.^14^ A Danish study found that low TTR within 6 months after the surgery was associated with a higher long-term thromboembolic risk^16^; however, the used TTR threshold of 70% classified majority of the patients to the ‘low-quality’ anticoagulation group. A Swedish study found no significant correlation between TTR and thromboembolic complications,^15^ while another study by the same author reported a significant correlation between low TTR and thromboembolic events,^20^ though the endpoint also included venous thromboses. Overall, there is a need for analyses exploiting probability curves for optimal cut-off point identification, more advanced TTR calculations, and an adequate consideration of missing INR data. The TTR calculation method presented here facilitates the identification of patients at highest stroke risk, allowing for more targeted preventive measures.

A significant proportion of strokes occurred within 60 days following an invasive procedure. As previously reported, perioperative interruption of OAC is common in patients who experience a postoperative stroke.^21^ While avoiding perioperative interruption of OAC therapy is critical in patients with AF, it is exceptionally important for those with mechanical valve prosthesis.

Myocardial infarctions were of particular interest due to the high incidence of CAD-related deaths following mechanical AVR.^6^ Our study found no significant correlation between low TTR and the occurrence of MI, nor any significant linear decline in TTR during the months preceding the event. These findings suggest that MI cannot be prevented solely through meticulous INR-guided VKA therapy. Instead, the focus should be on intensive primary and secondary prevention of atherosclerotic vascular disease in patients with mechanical AVR.

### Mortality

Almost one-third of the patients died during the follow-up period. Mortality was notably high, considering these patients are relatively young and expected to outlive the typical 10–15-year lifespan of surgical and transcatheter biological valve prostheses.^22^ The prognosis in this study was significantly worse than previously reported,^3–6,23^ although Zulkifly *et al*.^24^ reported a similar mortality. The definitive causes of the high mortality remain unclear, but it has been suggested that the suboptimal flow profile of mechanical prostheses subjects the left ventricle to persistent strain, fibrosis, and reduced coronary flow reserve.^3^ This is supported by the finding that CAD was the leading underlying cause of death (see Supplementary material online, *Table S7*), although only 56% of the patients who died from CAD had pre-existing CAD prior to the operation. Furthermore, patients who died during follow-up exhibited a higher incidence of well-known cardiovascular risk factors, which further supports this hypothesis.

Nearly half of the patients with the mean TTR below 80% died during 10-year follow-up, while those in the TTR ≥80% group had a relatively benign prognosis (*Figure 3B*). The association between low TTR and mortality in patients with mechanical heart valves has been previously reported.^15,24^ While part of the effect is mediated by general treatment adherence and other unexamined risk factors, the control of anticoagulation itself appears to play a significant role in the elevated risk, influenced by both ischaemic and bleeding events, which were also considered in this study.

### Time in therapeutic range values

In this study, the mean TTR was 81%, which is substantially higher than reported in previous studies,^14-16,24^ primarily due to the INR target range of 2.0–3.5 used in our analysis. This range is generally safe for the vast majority of patients with mechanical AVR. It is unlikely that short-term INR drops to 2.0–2.4 would significantly increase the risk of thromboembolism for patients with an INR target of 2.5–3.5 in the era of modern mechanical valves. Similarly, short-term INR peaks of 3.1–3.5 are unlikely to substantially raise the risk of bleeding in patients with an INR target of 2.0–3.0. For these reasons, we consider this INR range the most relevant for identifying patients at high thromboembolic or bleeding risk. In addition to the novel approach of incorporating this INR target range into TTR calculations, our findings suggest that an optimal TTR cut-off for patients with mechanical AVR is 80%. This threshold provides optimal true positive rate for clinical application and should be exploited in future research. Moreover, although the most recent ESC/EACTS and ACC/AHA guidelines recommend targeting a median INR value rather than a range,^1,2^ future guidelines should incorporate the TTR calculation method with an INR target of 2.0–3.5 and the cut-off as a clinical tool to evaluate the quality of VKA therapy in this patient population.

### Future concepts

The present results indicate that patients undergoing mechanical AVR are at high risk of adverse events, especially clinically significant bleeding episodes and mortality, and that the patients at highest risk can be identified with TTR calculations. Moreover, significant proportion of the adverse events could presumably be prevented with better anticoagulation control. For the time being, effective interventions on the quality of anticoagulation treatment are a disregarded possibility to improve patient outcome. In the present era of modern technology, INR controls and VKA doses are still largely determined by healthcare workers, although computer-aided dosing would perform better.^25^ Home monitoring is an underutilized method that improves the quality of the treatment,^26^ and besides automatic dosage determinations, the integration with smart devices would allow effective TTR monitoring and automatic alerts to healthcare units when risk zones or significant fluctuation are detected. Naturally, thorough patient education including dietary advice and information on drug interactions with warfarin should be highly emphasized and repeated when INR instability is detected. The education should be delivered through pharmacist-led interventions, which have been shown to improve patient outcomes—particularly by reducing bleeding episodes.^27^ Whichever method is chosen, the potential for better anticoagulation control to improve patient outcome seems enormous and the possibilities in this field are continuously evolving.

### Strengths and limitations

A major strength of our study is its comprehensive, nationwide data collection. The data were combined from all available nationwide registries, encompassing hospital admissions, outpatient healthcare visits, mortality records, prescription information, and laboratory results. This approach ensured that the data were as extensive and inclusive as possible within a registry-based framework. However, the study is entirely reliant on registry data and is subject to the general limitations of such an approach. Consequently, information on factors such as smoking, alcohol consumption, height, and weight was unavailable, and the accuracy of the data is dependent on how it was recorded. Fortunately, the Finnish special care register (HILMO), in particular, has a long-standing reputation for high-quality and well-validated data, with e.g. the stroke diagnoses reliably registered.^28,29^ While the multivariable analyses were adjusted for known thromboembolic and bleeding risk factors, the potential impact of unmeasured confounders and residual confounding cannot be excluded. Moreover, data on INR home monitoring and its recorded values were unavailable, making it impossible to assess its impact on TTR values or study endpoints. In addition to INR home monitoring, INR values from certain local laboratories were also unavailable, which may affect the generalizability of the results. As the less specific AF diagnosis code was commonly used during the study period, further analysis of AF subtypes was not feasible. Additionally, the study included only patients with AF diagnosed either before or after the index operation, so the findings may not be directly generalizable to all patients with mechanical AVR. However, the majority of patients with mechanical AVR either have pre-existing AF or develop AF during long-term follow-up.^7^ Therefore, this analysis provides a relatively inclusive overview of patients with mechanical AVR in general.

## Conclusions

This retrospective nationwide cohort study demonstrated that adverse events, particularly clinically significant bleeding episodes, are common, and mortality is high in patients with AF undergoing mechanical AVR. Suboptimal TTR appears to identify patients at high risk of bleeding, ischaemic events, and death. Greater efforts are needed to optimize anticoagulation management to improve the otherwise poor prognosis in this patient population following mechanical AVR.

## Supplementary Material

qcaf028_Supplementary_Data

## Data Availability

Based on the contracts with the Finnish registries, the data are not available for sharing.
