# Supplementary material for: Quality of anticoagulation and outcomes after mechanical aortic valve replacement in patients with atrial fibrillation: a nationwide cohort study
Source: Eur Heart J Qual Care Clin Outcomes. 2025 May 14;11(5):654–64. doi: 10.1093/ehjqcco/qcaf028 (PMC12342859; doi:10.1093/ehjqcco/qcaf028)
Supplement: qcaf028_Supplementary_Data [file qcaf028_supplementary_data.zip › FinACAF_supplementary_material_unmarked.docx]

**Supplementary Material**

**Supplementary Table 1.** Definitions of the comorbidities and outcomes.

**Supplementary Table 2.** Baseline characteristics of patients with and without composite endpoint after mechanical aortic valve replacement (AVR) and univariable Fine-Gray subdistribution hazard ratios.

**Supplementary Table 3.** Number of patients with comprehensive INR data available. Patients with the episode in question within a 30-day postoperative blanking period or follow-up time under 60 days were excluded.

**Supplementary Table 4.** Baseline characteristics of patients with TTR ≥80% and <80% after mechanical aortic valve replacement (AVR).

**Supplementary Table 5.** Baseline characteristics of patients with and without intracranial hemorrhage (ICH) after mechanical aortic valve replacement (AVR) and univariable Fine-Gray subdistribution hazard ratios.

**Supplementary Table 6.** Baseline characteristics of patients with and without myocardial infarction (MI) after mechanical aortic valve replacement (AVR) and univariable Fine-Gray subdistribution hazard ratios.

**Supplementary Table 7.** The reported underlying diagnoses of death after mechanical aortic valve replacement (AVR).

**Supplementary Figure 1.** Flow-chart of the patient selection process.

**Supplementary Figure 2.** The distribution of all International Normalized Ratio (INR) values during follow-up.

**Supplementary Figure 3.** The distribution of rolling daily 60-day Time in Therapeutic Range (TTR) values with International Normalized Ratio (INR) target 2.0–3.5.

**Supplementary Figure 4.** Cumulative incidence of composite endpoint after mechanical aortic valve replacement (AVR) (A), International Normalized Ratio (INR) at the time of the event (B), and the unadjusted cumulative incidence stratified by Time in Therapeutic Range (TTR) (INR target 2.0–3.5) <80% and ≥80% (C).

**Supplementary Figure 5.** Evolution of International Normalized Ratio (INR) values preceding the composite endpoint (A), any significant bleeding (B), intracranial hemorrhage (ICH) (C), ischemic stroke (D), and myocardial infarction (MI) (E). Data points represent individual patients’ raw INR values, while the line depicts the slope estimate from the Linear Mixed Model (LMM).

**Supplementary Figure 6.** Cumulative incidence of intracranial hemorrhage (ICH) after mechanical aortic valve replacement (AVR) (A), International Normalized Ratio (INR) at the time of the event (B), and the unadjusted cumulative incidence stratified by Time in Therapeutic Range (TTR) (INR target 2.0–3.5) <80% and ≥80% (C).

**Supplementary Figure 7.** Flow chart of the timing of ischemic strokes in relation to the initial atrial fibrillation (AF) diagnosis and mechanical aortic valve replacement (AVR).

**Supplementary Figure 8.** Cumulative incidence of myocardial infarction (MI) after mechanical aortic valve replacement (AVR) (A), International Normalized Ratio (INR) at the time of the event (B), and the unadjusted cumulative incidence stratified by Time in Therapeutic Range (TTR) (INR target 2.0–3.5) <80% and ≥80% (C).

**Supplementary Table 1**. Definitions of the comorbidities and outcomes.

|  | ICD-10 | ICPC-2 | Reimbursement code | ATC code |
| --- | --- | --- | --- | --- |
| **Outcomes** |  |  |  |  |
| Any clinically significant bleeding | D50.0, D62, D68.3, I60-I62, I85.0, I86.4, J94.2, K22.1, K22.3, K22.6, K25.0, K25.2, K25.4, K25.6, K26.0, K26.2, K26.4, K26.6, K27.0, K27.2, K27.4, K27.6, K28.0, K28.2, K28.4, K28.6, K29.0, K62.5, K63.1, K63.3, K92.0-K92.2, N02, R04, R31, R58, S06.4-S06.6 |  |  |  |
| Gastrointestinal bleeding | D50.0, I85.0, I86.4, K22.1, K22.3, K22.6, K25.0, K25.2, K25.4, K25.6, K26.0, K26.2, K26.4, K26.6, K27.0, K27.2, K27.4, K27.6, K28.0, K28.2, K28.4, K28.6, K29.0, K62.5, K63.1, K63.3, K92.0-K92.2 |  |  |  |
| Intracranial bleeding | I60-I62, S06.4-S06.6 |  |  |  |
| Myocardial infarction | I21-I22 |  |  |  |
| Ischemic stroke | I63 |  |  |  |
| **Comorbidities** |  |  |  |  |
| Aortic valve stenosis | I35.0, I35.2, I06.0, I06.2 |  |  |  |
| Aortic valve insufficiency | I35.1, I35.2, I06.1, I06.2 |  |  |  |
| Previous infective endocarditis | B37.6, I33, I38, I39.8 |  |  |  |
| Hypertension | I10-I15 | K85, K86, K87 | 205 | C03A, C03B, C03DB, C03EA, C07A, C08CA, C08D, C09 |
| Dyslipidemia | E78 | T93 | 206 | C10 |
| Heart failure | I50, I11.0, I13.0, I13.2 | K77 | 201 |  |
| Diabetes | E10-E14 | T89, T90 | 103, 215 | A10 |
| Previous stroke | I63, I64, I69.3-I69.8 | K90 |  |  |
| Previous transient ischemic attack | G45 | K89 |  |  |
| Any previous bleeding | D50.0, D62, D68.3, I60-I62, I85.0, I86.4, J94.2, K22.1, K22.3, K22.6, K25.0, K25.2, K25.4, K25.6, K26.0, K26.2, K26.4, K26.6, K27.0, K27.2, K27.4, K27.6, K28.0, K28.2, K28.4, K28.6, K29.0, K62.5, K63.1, K63.3, K92.0-K92.2, N02, R04, R31, R58, S06.4-S06.6 |  |  |  |
| Alcohol abuse | F10 | P15, P16 |  |  |
| Renal failure or dialysis | N18, Z49 |  |  |  |
| Liver cirrhosis or failure | K70.2-K70.4, K71.7, K71.8, K72, K74 |  |  |  |
| Coronary artery disease | I21-I25 | K74, K75, K76 | 206 |  |
| Prior myocardial infarction | I21-I22 |  |  |  |
| Psychiatric disorder | F04-F99 |  |  |  |
| Vascular disease | I20-I25, I65-I66, I67.2, I70 | K74, K75, K76, K91, K92 | 206 |  |
| Abbreviations: ATC, anatomic therapeutic chemical; ICD-10, International Classification of Diseases, Tenth Revision; ICPC-2, International Classification of Primary Care, Second Edition. | | | | |

**Supplementary Table 2.** Baseline characteristics of patients with and without composite endpoint after mechanical aortic valve replacement (AVR) and univariable Fine-Gray subdistribution hazard ratios.

|  | No composite endpoint  n=712 | Composite endpoint  n=374 | Hazard ratio  (95% CI) | P-value |
| --- | --- | --- | --- | --- |
| Age, years | 61.5 [54.7–67.1] | 65.2 [58.7–70.2] | 1.25 (1.12–1.40) | <0.001 |
| Year of index surgery | 2008 [2006–2012] | 2007 [2005–2009] | 0.92 (0.81–1.04) | 0.170 |
| Female sex | 194 (27.2) | 100 (26.7) | 0.91 (0.72–1.14) | 0.410 |
| Aortic valve stenosis | 560 (78.7) | 284 (75.9) | 0.90 (0.71–1.14) | 0.370 |
| Aortic valve insufficiency | 432 (60.7) | 221 (59.1) | 1.00 (0.81–1.22) | 0.960 |
| Previous endocarditis | 41 (5.8) | 18 (4.8) | 1.00 (0.62–1.60) | 1.000 |
| Any vascular disease | 173 (24.3) | 122 (32.6) | 1.35 (1.09–1.67) | 0.007 |
| Coronary artery disease | 168 (23.6) | 113 (30.2) | 1.28 (1.03–1.60) | 0.027 |
| Diabetes | 170 (23.9) | 104 (27.8) | 1.41 (1.12–1.77) | 0.003 |
| Dyslipidemia | 347 (48.7) | 200 (53.5) | 1.17 (0.96–1.44) | 0.120 |
| Heart failure | 154 (21.6) | 97 (25.9) | 1.23 (0.98–1.55) | 0.075 |
| Hypertension | 583 (81.9) | 308 (82.4) | 1.15 (0.89–1.50) | 0.290 |
| Prior TIA | 24 (3.4) | 12 (3.2) | 1.34 (0.73–2.45) | 0.340 |
| Abnormal liver function | 4 (0.6) | 1 (0.3) | 1.23 (0.173–8.77) | 0.840 |
| Abnormal renal function | 4 (0.6) | 1 (0.3) | 0.47 (0.064–3.46) | 0.460 |
| Alcohol use disorder | 15 (2.1) | 7 (1.9) | 1.12 (0.57–2.20) | 0.740 |
| Psychiatric disorder | 43 (6.0) | 19 (5.1) | 1.20 (0.77–1.87) | 0.430 |
| Modified HAS-BLED score | 1.0 [1.0–2.0] | 1.0 [1.0–2.0] | 1.15 (1.04–1.27) | 0.006 |
| CHA_2_DS_2_-VA score | 2.0 [1.0–3.0] | 3.0 [2.0–4.0] | 1.27 (1.15–1.41) | <0.001 |
| Values denote n (%) or median [25^th^ – 75^th^ percentile]. Standardized hazard ratio for continuous variables. Abbreviations: CHA_2_DS_2_-VA, congestive heart failure, hypertension, age ≥75 years, diabetes, history of stroke or TIA, vascular disease, age 65-74 years; CI: confidence interval; modified HAS-BLED score, hypertension, abnormal renal or liver function, prior stroke, bleeding history, age >65 years, alcohol abuse, concomitant antiplatelet/NSAIDs (no labile INR, max score 8); TIA, transient ischemic attack. | | | | |

**Supplementary Table 3.** Number of patients with comprehensive INR data available. Patients with the episode in question within a 30-day postoperative blanking period or follow-up time under 60 days were excluded.

|  | Comprehensive INR data available |
| --- | --- |
| AVR – end of follow-up: |  |
| All patients | 675/1046 (64.5%) |
| Composite endpoint | 230/349 (65.9%) |
| No composite endpoint | 427/677 (63.1%) |
| Clinically significant bleeding | 190/287 (66.2%) |
| No clinically significant bleeding | 477/750 (63.6%) |
| ICH | 39/58 (67.2%) |
| No ICH | 636/988 (64.4%) |
| Ischemic stroke | 79/114 (69.3%) |
| No ischemic stroke | 586/921 (63.6%) |
| MI | 45/75 (60.0%) |
| No MI | 625/964 (64.8%) |
| Dead | 160/287 (55.7%) |
| Alive | 515/759 (67.9%) |
| AVR – event: |  |
| Composite endpoint | 155/349 (44.4%) |
| Clinically significant bleeding | 131/287 (45.6%) |
| ICH | 30/58 (51.7%) |
| Ischemic stroke | 53/114 (46.5%) |
| MI | 31/75 (41.3%) |
| Values denote n (%). AVR, aortic valve replacement; ICH, intracranial hemorrhage; INR, international normalized ratio; MI, myocardial infarction. | |

**Supplementary Table 4.** Baseline characteristics of patients with TTR ≥80% and <80% after mechanical aortic valve replacement (AVR).

|  | TTR ≥80%  n=438 | TTR <80%  n=237 | P-value |
| --- | --- | --- | --- |
| Age, years | 62.0 [56.3–67.2] | 61.7 [54.8–68.1] | 0.900 |
| Year of index surgery | 2008 [2005–2012] | 2008 [2005–2011] | 0.651 |
| Female sex | 110 (25.1) | 65 (27.4) | 0.574 |
| Aortic valve stenosis | 344 (78.5) | 177 (74.7) | 0.297 |
| Aortic valve insufficiency | 284 (64.8) | 132 (55.7) | 0.025 |
| Previous endocarditis | 16 (3.7) | 18 (7.6) | 0.040 |
| Any vascular disease | 114 (26.0) | 78 (32.9) | 0.071 |
| Coronary artery disease | 111 (25.3) | 75 (31.6) | 0.097 |
| Diabetes | 106 (24.2) | 74 (31.2) | 0.060 |
| Dyslipidemia | 214 (48.9) | 129 (54.4) | 0.193 |
| Heart failure | 91 (20.8) | 58 (24.5) | 0.313 |
| Hypertension | 360 (82.2) | 198 (83.5) | 0.736 |
| Prior TIA | 13 (3.0) | 10 (4.2) | 0.527 |
| Abnormal liver function | 1 (0.2) | 2 (0.8) | 0.283 |
| Abnormal renal function | 1 (0.2) | 2 (0.8) | 0.283 |
| Alcohol use disorder | 7 (1.6) | 7 (3.0) | 0.264 |
| Psychiatric disorder | 23 (5.3) | 15 (6.3) | 0.685 |
| Modified HAS-BLED score | 1.0 [1.0–2.0] | 1.0 [1.0–2.0] | 0.523 |
| CHA_2_DS_2_-VA score | 2.0 [1.0–3.0] | 2.0 [1.0–3.0] | 0.019 |
| Income quartiles: |  |  | 0.381 |
| 1st | 82 (18.7) | 57 (24.1) |  |
| 2nd | 119 (27.2) | 56 (23.6) |  |
| 3rd | 121 (27.6) | 61 (25.7) |  |
| 4th | 116 (26.5) | 63 (26.6) |  |
| Educational categories: |  |  | 0.195 |
| 1st | 175 (40.0) | 110 (46.4) |  |
| 2nd | 148 (33.8) | 77 (32.5) |  |
| 3rd | 115 (26.3) | 50 (21.1) |  |
| Values denote n (%), mean (standard deviation), or median [25^th^ – 75^th^ percentile], as appropriate. Abbreviations: CHA_2_DS_2_-VA, congestive heart failure, hypertension, age ≥75 years, diabetes, history of stroke or TIA, vascular disease, age 65-74 years; modified HAS-BLED score, hypertension, abnormal renal or liver function, prior stroke, bleeding history, age >65 years, alcohol abuse, concomitant antiplatelet/NSAIDs (no labile INR, max score 8); TIA, transient ischemic attack; TTR, time in therapeutic range. | | | |

**Supplementary Table 5.** Baseline characteristics of patients with and without intracranial hemorrhage (ICH) after mechanical aortic valve replacement (AVR) and univariable Fine-Gray subdistribution hazard ratios.

|  | No ICH  n=1027 | ICH  n=59 | Hazard ratio  (95% CI) | P-value |
| --- | --- | --- | --- | --- |
| Age, years | 62.7 [55.8–68.3] | 64.2 [58.4–70.2] | 1.15 (0.86–1.53) | 0.350 |
| Year of index surgery | 2008 [2005–2011] | 2007 [2006–2008] | 1.12 (0.83– 1.5) | 0.460 |
| Female sex | 276 (26.9) | 18 (30.5) | 1.13 (0.65–1.96) | 0.670 |
| Aortic valve stenosis | 796 (77.5) | 48 (81.4) | 1.28 (0.67–2.46) | 0.460 |
| Aortic valve insufficiency | 620 (60.4) | 33 (55.9) | 0.87 (0.52–1.45) | 0.590 |
| Previous endocarditis | 55 (5.4) | 4 (6.8) | 1.48 (0.53– 4.1) | 0.450 |
| Any vascular disease | 280 (27.3) | 15 (25.4) | 0.88 (0.49–1.57) | 0.660 |
| Coronary artery disease | 266 (25.9) | 15 (25.4) | 0.94 (0.52–1.69) | 0.840 |
| Diabetes | 262 (25.5) | 12 (20.3) | 0.86 (0.46–1.62) | 0.650 |
| Dyslipidemia | 519 (50.5) | 28 (47.5) | 0.89 (0.53–1.47) | 0.640 |
| Heart failure | 238 (23.2) | 13 (22.0) | 0.95 (0.51–1.75) | 0.860 |
| Hypertension | 842 (82.0) | 49 (83.1) | 1.17 (0.60–2.31) | 0.650 |
| Prior TIA | 34 (3.3) | 2 (3.4) | 1.35 (0.32–5.64) | 0.690 |
| Abnormal liver function | 5 (0.5) | 0 | 0.00012 (0.000040– 0.00038) | <0.001 |
| Abnormal renal function | 5 (0.5) | 0 | 0.00012 (0.000049–0.00031) | <0.001 |
| Alcohol use disorder | 20 (1.9) | 2 (3.4) | 2.19 (0.54–8.88) | 0.270 |
| Psychiatric disorder | 59 (5.7) | 3 (5.1) | 1.19 (0.37–3.77) | 0.770 |
| Modified HAS-BLED score | 1.0 [1.0–2.0] | 1.0 [1.0–2.0] | 1.02 (0.78–1.32) | 0.910 |
| CHA_2_DS_2_-VA score | 2.0 [1.0–3.0] | 2.0 [1.0–3.0] | 1.02 (0.8– 1.3) | 0.860 |
| Values denote n (%) or median [25^th^ – 75^th^ percentile]. Standardized hazard ratio for continuous variables. Abbreviations: CHA_2_DS_2_-VA, congestive heart failure, hypertension, age ≥75 years, diabetes, history of stroke or TIA, vascular disease, age 65-74 years; CI: confidence interval; modified HAS-BLED score, hypertension, abnormal renal or liver function, prior stroke, bleeding history, age >65 years, alcohol abuse, concomitant antiplatelet/NSAIDs (no labile INR, max score 8); ICH, intracranial hemorrhage; TIA, transient ischemic attack. | | | | |

**Supplementary Table 6.** Baseline characteristics of patients with and without myocardial infarction (MI) after mechanical aortic valve replacement (AVR) and univariable Fine-Gray subdistribution hazard ratios.

|  | No MI  n=1004 | MI  n=82 | Hazard ratio  (95% CI) | P-value |
| --- | --- | --- | --- | --- |
| Age, years | 62.6 [55.8–68.0] | 66.3 [58.3–71.2] | 1.18 (0.93–1.49) | 0.170 |
| Year of index surgery | 2008 [2005–2012] | 2006 [2005–2008] | 0.81 (0.61–1.07) | 0.140 |
| Female sex | 277 (27.6) | 17 (20.7) | 0.64 (0.38–1.09) | 0.099 |
| Aortic valve stenosis | 776 (77.3) | 68 (82.9) | 1.45 (0.82–2.57) | 0.200 |
| Aortic valve insufficiency | 607 (60.5) | 46 (56.1) | 0.88 (0.57–1.36) | 0.560 |
| Previous endocarditis | 53 (5.3) | 6 (7.3) | 1.67 (0.72–3.87) | 0.230 |
| Any vascular disease | 260 (25.9) | 35 (42.7) | 2.02 (1.3–3.12) | 0.002 |
| Coronary artery disease | 247 (24.6) | 34 (41.5) | 2.07 (1.34–3.22) | 0.001 |
| Diabetes | 254 (25.3) | 20 (24.4) | 1.12 (0.68–1.86) | 0.650 |
| Dyslipidemia | 495 (49.3) | 52 (63.4) | 1.76 (1.12–2.76) | 0.013 |
| Heart failure | 233 (23.2) | 18 (22.0) | 0.94 (0.56–1.59) | 0.820 |
| Hypertension | 820 (81.7) | 71 (86.6) | 1.6 (0.85–3.01) | 0.150 |
| Prior TIA | 33 (3.3) | 3 (3.7) | 1.51 (0.47–4.87) | 0.490 |
| Abnormal liver function | 5 (0.5) | 0 | 0.00012 (0.000041–0.00037) | <0.001 |
| Abnormal renal function | 4 (0.4) | 1 (1.2) | 2.53 (0.36–17.9) | 0.350 |
| Alcohol use disorder | 20 (2.0) | 2 (2.4) | 1.6 (0.39–6.52) | 0.510 |
| Psychiatric disorder | 58 (5.8) | 4 (4.9) | 1.15 (0.42–3.12) | 0.790 |
| Modified HAS-BLED score | 1.0 [1.0–2.0] | 2.0 [1.0–2.0] | 1.29 (1.05–1.59) | 0.017 |
| CHA_2_DS_2_-VA score | 2.0 [1.0–3.0] | 2.0 [2.0–3.0] | 1.28 (1.07–1.54) | 0.008 |
| Values denote n (%) or median [25^th^ – 75^th^ percentile]. Standardized hazard ratio for continuous variables. Abbreviations: CHA_2_DS_2_-VA, congestive heart failure, hypertension, age ≥75 years, diabetes, history of stroke or TIA, vascular disease, age 65-74 years; CI: confidence interval; modified HAS-BLED score, hypertension, abnormal renal or liver function, prior stroke, bleeding history, age >65 years, alcohol abuse, concomitant antiplatelet/NSAIDs (no labile INR, max score 8); MI, myocardial infarction; TIA, transient ischemic attack. | | | | |

**Supplementary Table 7.** The reported underlying diagnoses of death after mechanical aortic valve replacement (AVR).

|  | n (%) |
| --- | --- |
| Coronary artery disease | 105 (32.6) |
| Aortic valve disease | 49 (15.2) |
| Cancer | 45 (14.0) |
| Neurological disease | 14 (4.3) |
| Pulmonary disease | 14 (4.3) |
| Accident or suicide | 11 (3.4) |
| Other valve disease | 10 (3.1) |
| Any bleeding | 9 (2.8) |
| Cardiomyopathy | 9 (2.8) |
| Ischemic stroke | 8 (2.5) |
| Aortic aneurysm or dissection | 5 (1.6) |
| Sepsis or endocarditis | 5 (1.6) |
| Bowel disease | 4 (1.2) |
| Diabetes | 4 (1.2) |
| Hypertension | 4 (1.2) |
| Liver cirrhosis | 4 (1.2) |
| Dementia | 3 (0.9) |
| Atrial fibrillation | 2 (0.6) |
| Other vascular disease | 2 (0.6) |
| Other | 13 (4.0 |
| Unknown | 2 (0.6) |
| Values denote n (%). AVR, aortic valve replacement. | |

**Supplementary Figure 1.** Flow-chart of the patient selection process.

**Supplementary Figure 2.** The distribution of all International Normalized Ratio (INR) values during follow-up.


**Supplementary Figure 3.** The distribution of rolling daily 60-day Time in Therapeutic Range (TTR) values with International Normalized Ratio (INR) target 2.0–3.5.

**Supplementary Figure 4.** Cumulative incidence of composite endpoint after mechanical aortic valve replacement (AVR) (A), International Normalized Ratio (INR) at the time of the event (B), and the unadjusted cumulative incidence stratified by Time in Therapeutic Range (TTR) (INR target 2.0–3.5) <80% and ≥80% (C).

**Supplementary Figure 5.** Evolution of International Normalized Ratio (INR) values preceding the composite endpoint (A), any significant bleeding (B), intracranial hemorrhage (ICH) (C), ischemic stroke (D), and myocardial infarction (MI) (E). Data points represent individual patients’ raw INR values, while the line depicts the slope estimate from the Linear Mixed Model (LMM).

**Supplementary Figure 6.** Cumulative incidence of intracranial hemorrhage (ICH) after mechanical aortic valve replacement (AVR) (A), International Normalized Ratio (INR) at the time of the event (B), and the unadjusted cumulative incidence stratified by Time in Therapeutic Range (TTR) (INR target 2.0–3.5) <80% and ≥80% (C).


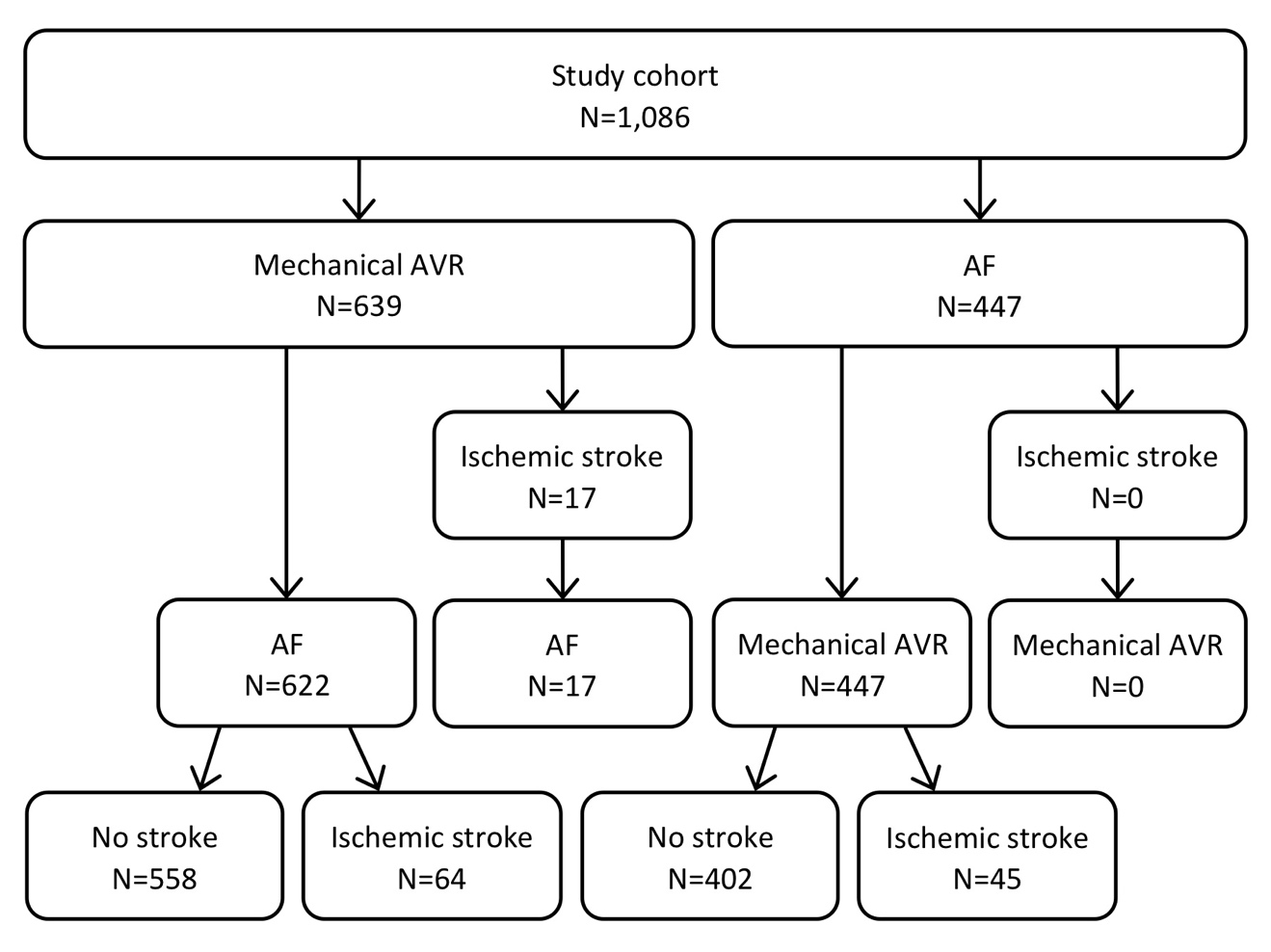
**Supplementary Figure 7.** Flow chart of the timing of ischemic strokes in relation to the initial atrial fibrillation (AF) diagnosis and mechanical aortic valve replacement (AVR).

**Supplementary Figure 8.** Cumulative incidence of myocardial infarction (MI) after mechanical aortic valve replacement (AVR) (A), International Normalized Ratio (INR) at the time of the event (B), and the unadjusted cumulative incidence stratified by Time in Therapeutic Range (TTR) (INR target 2.0–3.5) <80% and ≥80% (C).
